# Point-of-care testing with Xpert HPV for single-visit, screen-and-treat for cervical cancer prevention: a demonstration study in South Africa

**DOI:** 10.1038/s41598-023-43467-2

**Published:** 2023-09-27

**Authors:** Lynette Denny, Rakiya Saidu, Rosalind Boa, Nomonde Mbatani, Delivette Castor, Jennifer Moodley, Louise Kuhn

**Affiliations:** 1 Department Obstetrics and Gynaecology, Faculty of Health Sciences, University of Cape Town, Cape Town, South Africa; 2 Department Obstetrics and Gynaecology, South African Medical Research Council, Gynaecology Cancer Research Centre, Faculty of Health Sciences, University of Cape Town, Cape Town, South Africa; 3 Department Epidemiology, Mailman School of Public Health, Columbia University Irving Medical Center, New York, USA; 4 Division of Infectious Diseases, Department of Medicine, Vagelos College of Physicians and Surgeons, Columbia University Irving Medical Center, New York, USA; 5 Cancer Research Initiative and School of Public Health, Faculty of Health Sciences, University of Cape Town, Cape Town, South Africa; 6 Gertrude H. Sergievsky Center, Vagelos College of Physicians and Surgeons, Columbia University Irving Medical Center, 630 W 168th Street, New York, NY 10032 USA

**Keywords:** Cancer, Health care

## Abstract

Human papillomavirus (HPV)-based screen-and-treat (SAT) is recommended but implementation presents operational challenges. We implemented HPV-SAT at a research site in Khayelitsha, South Africa, screening 3062 women aged 30–65 years (44% women living with HIV [WHIV]). All were screened using point-of-care Xpert HPV and almost all received their HPV results on the same day. HPV-positivity occurred in 41.5% of WHIV and 17.4% of women without HIV (WNoH) reducing to 26.2% in WHIV and 10.4% in WNoH applying treatment eligibility criteria based on high viral load in the channels detecting HPV16, 18, 45, 16, 18, 31, 33, 35, 52, 58. Among those eligible for treatment, 91.3% were considered suitable for ablative therapy, and 94.6% underwent thermal ablation on the same day, with no serious adverse events. Twelve months later, 39.0% of WHIV and 65.2% of WNoH treated with ablative therapy were clear of HPV. In women who were HPV-positive but ineligible for treatment, 19.1% and 12.9% had histologically-confirmed cervical intraepithelial neoplasia grade 2 or worse (CIN2+) at 12 months. SAT programs need to weigh trade-offs between overtreatment versus delayed or no treatment for women who test positive for HPV. Treatment modalities for precancerous lesions need to be improved.

## Introduction

Cervical cancer is one of the most preventable cancers due to the possibility of detecting and treating during the precancerous phase before the development of invasive disease. Yet, global inequality in access to quality health care has resulted in a highly-skewed prevalence, incidence, and mortality from cervical cancer^1^. Strengthening cervical cancer screening programs in Low and Middle Income Countries (LMICs) is essential to address global inequity^2^. Weak linkage between the screening test and follow-up treatment threatens the goals of the World Health Organization (WHO) elimination of cervical cancer as a public health problem agenda. Recognizing this critical gap in program efficiencies, WHO endorsed a simplified approach to screening called screen-and-treat (SAT). In SAT, a positive screening test result, a human papillomavirus (HPV) test, or visual inspection with acetic acid (VIA), identifies those at highest risk of precursor lesions that can be treated immediately—reducing attrition introduced by referral to colposcopy and histological confirmation. Although known to be safe and effective, including in women living with HIV (WHIV)^3–6^, much remains unknown about the implementation and scale-up HPV-SAT in real-world primary health care settings.

Some have questioned the desirability of HPV-SAT for populations with high HIV prevalence. WHIV have high rates of HPV co-infection, but not all will progress to cancer. Treating all WHIV who are HPV co-infected burdens the health care system and imposes unnecessary discomfort on women. Recently, our group proposed a modification to the interpretation of the Xpert HPV assay that reduces the number of women defined as “positive”, and thus reducing over-treatment, without substantial compromise in sensitivity^7,8^. This is done by restricting the definition of a positive test to a subset of HPV genotypes at higher viral load thresholds^7,8^. In addition, Xpert HPV can be used at the point-of-care (POC). POC testing is desirable because women can receive their screening results and be treated at a single visit. No additional reflex testing, triage tests, or new sampling is required.

Here we implemented this single-visit, HPV-SAT approach at a clinical research site in South Africa utilizing Xpert HPV, a POC assay, modifying the interpretation of this assay to define eligibility for treatment, and providing same-day ablative treatment for women who screened positive. Our goal was to evaluate real-life operational challenges in implementing this approach with task shifting to nurses and community health workers (CHW) to assess the feasibility of a broader roll-out.

## Results

### Study population

The study was implemented at a clinical research site co-located with a primary health care facility in Khayelitsha, outside Cape Town, South Africa. From May 2017 to September 2018, 3062 women, aged 30–65 years, residing in Khayelitsha were recruited into the study: 1346 (44.0%) were WHIV and 1716 (56.0%) were WNoH. Table 1 shows demographic information.

**Table 1:** Baseline characteristics of the 3062 women enrolled into the demonstration study between May 2017 to September 2018.

Characteristic	Total population	Women living with HIV	Women without HIV
Number of participants	3062	1346	1716
Age (years)			
Median (IQR)	42 (35, 50)	40 (35, 46)	43 (35, 52)
30–39	1308 (42.7%)	661 (49.1%)	647 (37.7%)
40–49	987 (32.2%)	486 (36.1%)	501 (29.2%)
50–59	615 (20.1%)	171 (12.7%)	444 (25.9%)
60+	152 (5.0%)	28 (2.1%)	124 (7.2%)
Age at first pregnancy			
Median (IQR)	20 (18, 24)	20 (18, 24)	20 (18, 24)
Never pregnant	132 (4.3%)	71 (5.3%)	61 (2.0%)
≤ 19 years	1210 (39.5%)	523 (38.9%)	687 (40.0%)
> 19 years	1720 (56.2%)	752 (55.9%)	968 (56.4%)
Parity			
Median (IQR)	2 (2, 3)	2 (1, 3)	2 (2, 4)
None	172 (5.6%)	90 (6.7%)	82 (4.8%)
1	555 (18.1%)	268 (19.9%)	287 (16.7%)
2–4	2058 (67.2%)	899 (66.8%)	1159 (67.5%)
5–8	277 (9.0%)	89 (6.6%)	188 (11.0%)
Primary contraception			
None	1592 (52.0%)	708 (52.6%)	884 (51.5%)
Tubal ligation	553 (18.1%)	207 (15.4%)	346 (20.2%)
Combined OC	22 (0.7%)	3 (0.2%)	19 (1.1%)
DepoProvera	510 (16.7%)	275 (20.4%)	235 (13.7%)
Nuristerate	122 (4.0%)	53 (3.9%)	69 (4.0%)
Implanon	120 (4.0%)	23 (1.7%)	97 (5.6%)
Oral progestogen	11 (0.4%)	3 (0.2%)	8 (0.5%)
IUCD	131 (4.3%)	73 (5.4%)	58 (3.4%)
Mirena	1 (0.03%)	1 (0.07%)	0 (0.0%)
Condom use			
Always	850 (27.8%)	510 (37.9%)	340 (19.8%)
Sometimes	558 (18.2%)	301 (22.4%)	257 (15.0%)
Never	1654 (54.0%)	535 (39.8%)	1119 (65.2%)
Education			
None	42 (1.4%)	10 (0.7%)	32 (1.9%)
Only primary school	448 (14.6%)	167 (12.4%)	281 (16.4%)
Some high school	1682 (54.9%)	807 (60.0%)	875 (51.0%)
Grade 12/std 10	636 (20.8%)	274 (20.4%)	362 (21.1%)
Any tertiary	254 (8.3%)	88 (6.5%)	166 (9.7%)
Tobacco use			
Current	400 (13.1%)	216 (16.1%)	184 (10.7%)
Former	138 (4.5%)	74 (5.5%)	64 (3.7%)
Never	2524 (82.4%)	1056 (78.4%)	1468 (85.6%)
Co-morbidities			
On TB treatment	39 (1.3%)	37 (2.7%)	2 (0.1%)
Diabetic	258 (8.4%)	59 (4.4%)	199 (11.6%)
Other	815 (26.6%)	265 (19.7%)	550 (32.1%)
None	1950 (63.7%)	985 (73.2%)	965 (56.2%)
Currently on antiretroviral treatment			
Yes		1176 (87.4%)	
No		169 (12.6%)	
Know regimen			
Yes		1112 (82.6%)	
Don’t know		62 (4.6%)	
Not taking		172 (12.9%)	
Regimen			
Efavirenz/emtricitabine/tenofovir		961 (86.4%)	
Other		151 (13.6%)	
Most recent CD4 count			
N		N = 861	
Median (IQR)		463 (300, 666)	
Most recent viral load			
N		N = 594	
N (%) below 50 copies/ml		506 (85.2%)	

### Operational fidelity to the screen-and-treat cascade

**Figure 1:**
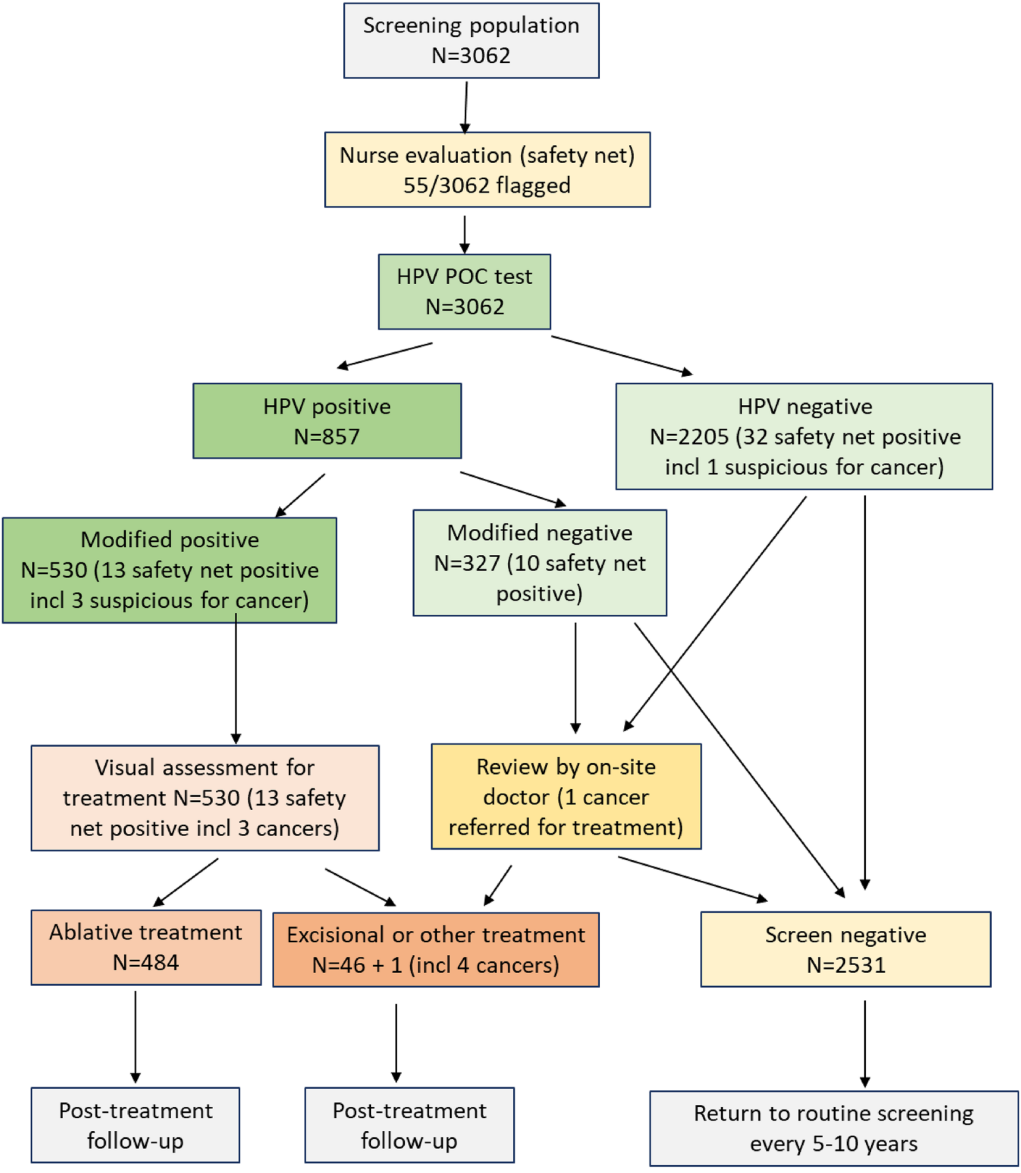
Schematic of the HPV-based screen-and-treat algorithm utilizing a modified definition of a positive HPV test to define eligibility for treatment as well as the numbers of study participants moving through each step in the screening cascade.

Figure 1 shows the screen-and-treat cascade. Before HPV testing, a study nurse performed a gynaecological examination, inspecting the lower anogenital tract and cervix without acetic acid. Fifty-five (1.8%) of 3062 women had conditions of concern, of which 4 were considered suspicious for cancer.

Next, a nurse-collected cervical sample was tested on-site with Xpert HPV. Overall, 857 (28.0%) women tested HPV-positive in one or more of the five channels at standard cut-offs (41.5% of WHIV and 17.4% of WNoH). Using the modified criteria, which involved restricting to three channels that detect HPV types 16, 18, 45, 31, 33, 35, 52, 58 and limiting further to more stringent cut-offs related to higher viral loads or multiple types, this number was reduced to 530 (17.3%) (26.2% of WHIV and 10.4% of WNoH).

Almost all (99.8%) women received their HPV results on the same day. Four of 6 women who did not receive their results on the same day, received the results the following day. Most (98.1%) assays yielded a valid result on the first run. The median waiting time from sample collection to receiving results was 1.52 h (IQR 1.35–1.80 h). Exactly, 473 (15.6%) and 44 (1.5%) women waited for more than 2 h and 3 h, respectively. Longer wait times were associated with not getting a valid result on the first run (Table 2).

**Table 2:** Results of point-of-care HPV testing with Xpert HPV to determine eligibility for treatment based on high viral load or multiple types of the channels detecting HPV 16, 18, 45, 31, 33, 35, 52, 58.

Variable	Total population	Women living with HIV	Women not living with HIV
Valid results obtained			
Valid on 1st run	3005 (98.1)	1320 (98.1)	1685 (98.2)
Valid on 2nd run	52 (1.7)	23 (1.7)	29 (1.7)
Valid on 3rd run	4	3	1
New sample needed	1	0	1
Woman received results on the same day			
Yes	3056 (99.8%)	1345 (99.9%)	1711 (99.7%)
No	6^a^ (0.2%)	1 (0.1%)	5 (0.3%)
Any high risk HPV (positive on any of the 5 channels)			
Yes	857 (28.0%)	559 (41.5%)	298 (17.4%)
No	2205 (72.0%)	787 (57.5%)	1418 (82.6%)
Positive for 16, 18, 45, 31, 33, 35, 52, 58 (3 channels)			
Yes	712 (23.3%)	462 (34.3%)	250 (14.6%)
No	2350 (76.7%)	884 (65.7%)	1466 (85.4%)
Multiples of the 3 channels			
Positive > 1 channel	127 (4.2)	108 (8.0)	19 (1.1)
Positive only 1 of the channels	585 (19.1%)	354 (26.3%)	231 (13.5%)
None of 3 channels positive	2350 (76.7%)	884 (65.7)	1466 (85.4%)
Meet modified HPV criteria			
High viral load or multiple 16, 18, 45, 31, 33, 35, 52, 58	530 (17.3%)	352 (26.2%)	178 (10.4%)
No	2532 (82.7%)	994 (73.8%)	1538 (89.6%)

^a^Three women had valid results on the first run and 1 on the second run but could not wait and two women had valid results on the third run or needed a repeat sample which was done the next day. 4/6 women received their results the next day.

Among 530 women who met modified criteria for treatment, 13/530 (2.5%) had conditions of concern on initial nurse examination and were reviewed by the on-site study doctor. Another 51/530 (9.6%) were considered unsuitable for immediate ablative therapy or the nurse was uncertain and were reviewed by the on-site doctor. On doctor review, 4/13 (3/10 WHIV; 1/3 WNoH) and 14/51 (12/39 WHIV; 2/12 WNoH) were referred back to the study nurse as suitable for ablative therapy. After this combined review, 484/530 (91.3%) were considered suitable for ablative treatment.

Nearly 95% (458/484) of women referred for thermal ablation of the transformation zone were treated on the same day by the study nurse. The remainder (n = 26) received ablative treatment later date (13 within a week, 12 within 8–14 days and one at 18 days post-screening). Only 4 complications from thermal ablation were noted during the procedure—3 women complained of moderate or excessive pain and one had mild vaginal wall burn.

At 6 and 12 months follow-up visits, 49 (11.6%) women reported that they had had complications following the procedure. Of these, 21 women had an unscheduled visit at the research site within 1–5 weeks post-treatment and an additional three women presented to a local clinic. All complications were minor and most were treated with antibiotics. No patients required up-referral, none were hospitalised, and none were severe enough to be reported at serious adverse events (Table 3).

**Table 3:** Suitability for ablative treatment among 530 women meeting modified HPV criteria for treatment and completion of ablative therapy and complications among 484 women deemed suitable for same-day ablative therapy.

	Total	Women living with HIV	Women not living with HIV
Meet modified HPV criteria for treatment			
Yes^a^	530 (17.3%)	352 (26.2%)	178 (10.4%)
No	2532	994	1538
If meet HPV criteria, considered suitable for ablative treatment			
Yes	484 (91.3)	318 (90.3)	166 (93.3)
No	46	34	12
If considered suitable, had same day treatment			
Yes	458 (94.6)	300 (94.3)	158 (95.2)
No	26	18	8
Any complications during the procedure			
Yes	4 (0.8%)	3 (0.9)	1 (0.6)
No	480	315	165
Patient reports of complications at 6 or 12 months			
Yes	49 (11.6%)	31^b^ (11.4%)	18^c^ (11.9%)
No	373	240	133
Protocol-defined adverse event (unscheduled visit to the site or to other health service)			
Yes	24 (5.7%)	13^d^ (4.8%)	11^e^ (7.3%)
No	398	258	140

^a^All of these women received their HPV results on the same day.

^b^WHIV: Excessive bleeding (8), excessive pain (4), infection/offensive discharge (21).

^c^WNoH: Excessive bleeding (8), excessive pain (2), infection/offensive discharge (12).

^d^WHIV: Offensive vaginal odour (1), bleeding (4), lower abdominal pain (1).

^e^WNoH: Offensive vaginal odour (8), bleeding (5), lower abdominal pain (2).

*The sum of numbers at the footnote is more that the numbers in the table, because some patients had more than one complication.

Of the 46/530 (8.7%) women who met modified HPV criteria for treatment but were unsuitable for immediate ablative treatment, 16 were treated with Premarin or antibiotics and scheduled for ablative therapy on-site after 6 weeks. Eleven were managed on-site, inclusive of 5 women who had Large Loop Excision of the Transformation Zone (LLETZ), 4 cone biopsies, and 2 cervical biopsies. Sixteen women were referred to a higher level of care (4 for large lesions > 75% of the cervix, 2 squamocolumnar junction (SCJ) not visible, 6 the whole cervix was not visualized, 3 for technical reasons and 1 woman with a bleeding disorder). Three women in this group who had also been identified as being suspicious for cancer on initial review were referred to a higher level of care. All three had confirmed histological diagnoses of squamous cell carcinoma (SCC).

In addition to the 13/55 women who had been flagged as having conditions of concern by the initial nurse evaluation and met modified HPV eligibility criteria (inclusive of the 3 cancers described above), 10/327 women who tested HPV-positive but did not meet eligibility criteria and 32/2205 women who tested HPV-negative were evaluated by the on-site study doctor. Of 42 in the latter two groups, one woman had histologically-confirmed SCC. This woman was classified as suspicious for cancer by the nurse on the initial exam and was in the HPV-negative group. She had an advanced cancer and was referred for chemoradiation but died from a pulmonary embolus before completing her treatment. She was retested using Xpert HPV and Roche Linear Array and both tests were negative for HPV. Of the 42 women, 5 had polyps removed, 24 were referred for a variety of gynecologic conditions and 12 were considered not to require further referral.

### Evaluation at 6 and 12 months

**Figure 2:**
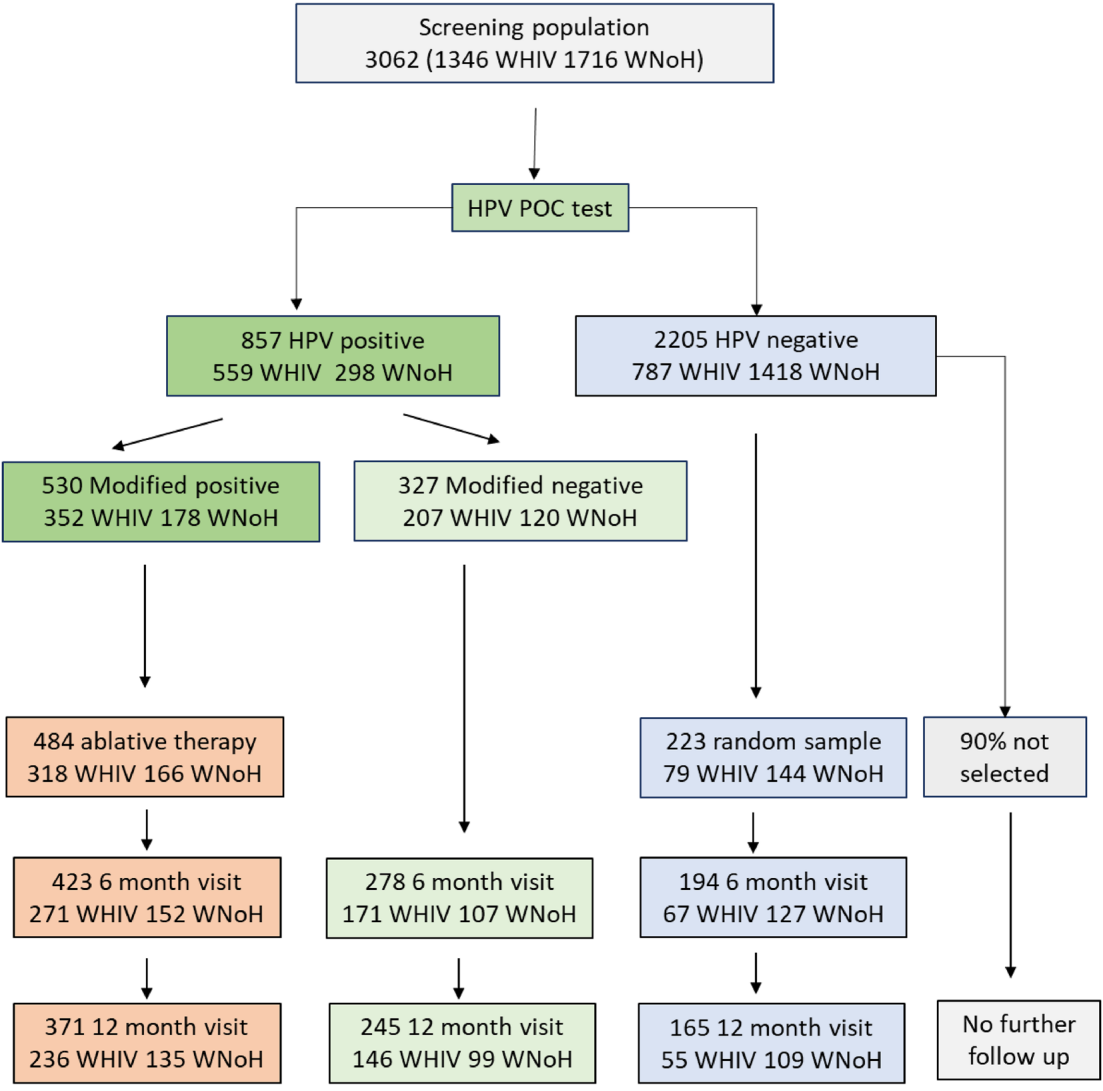
Schematic of the design of the follow-up evaluation including the numbers of women seen at each follow-up visit by HIV status.

All women who tested HPV-positive, whether or not they met modified treatment criteria, and a 10% random sample of women who tested HPV-negative were asked to return at 6 and 12 months (Fig. 2). At the follow-up visits, a clinician-taken cervical sample was re-tested on-site with Xpert HPV. All women underwent a colposcopic assessment and histological sampling.

#### Women who had undergone ablative therapy

The protocol defined the primary marker of ablative treatment effectiveness to be HPV clearance at 6 and 12 months. Histological sampling was not performed before treatment precluding evaluation of effectiveness of treatment based on this marker. Among 484 women suitable for ablative therapy, all of whom underwent this treatment, 423 (87.4%) were seen at 6 months and 371 (76.7%) at 12 months.

At 6 months, 29.2% of WHIV and 55.3% of WNoH and at 12 months, 39.0% of WHIV and 65.2% of WNoH had cleared all HPV. Clearance rates of the channels present at baseline were higher. At 12 months, 58.1% of WHIV and 68.9% of WNoH were negative for the channel they had tested positive for at baseline. Positivity for channels that were negative at baseline occurred in 19.5% of WHIV and 5.4% of WNoH. In terms of individual channel clearance rates, there were no differences between the channels (Table 4).

**Table 4:** Clearance of HPV among women eligible for immediate ablative therapy based on high viral load on select HPV genotypes clearance 6 and 12 months after ablative therapy.

	Women living with HIV	Women not living with HIV
N in group at baseline	318	166
HPV results at follow-up visits		
6 month visit		
N (Follow-up rate)	271 (85.2%)	152 (91.6%)
All 5 HPV channels		
Cleared all HPV	79 (29.2%)	84 (55.3%)
Cleared channel that was positive at baseline	138 (50.9%)	94 (61.8%)
New detection of channel not present at baseline	59 (21.8%)	10 (6.6%)
Individual channel clearance^a^		
Cleared HPV16	29/67 (43.3%)	31/48 (64.6%)
Cleared HPV18/45	28/69 (40.6%)	7/10 (70%)
Cleared P3 (HPV 31, 33, 35, 53, 58)	100/225 (44.4%)	67/110 (60.9%)
12 month visit		
N (follow-up rate)	236 (74.2%)	135 (81.3%)
All 5 HPV channels		
Cleared all HPV	92 (39.0%)	88 (65.2%)
Cleared channel that was positive at baseline	137 (58.1%)	93 (68.9%)
New detection of channel not present at baseline	46 (19.5%)	5 (5.4%)
Individual channel clearance^a^		
Cleared HPV16	31/58 (53.5%)	32/40 (80%)
Cleared HPV18/45	34/58 (58.6%)	7/9 (77.8%)
Cleared P3 (HPV 31, 33, 35, 53, 58)	102/196 (52.0%)	66/101 (65.4%)
Histology results at follow-up visits		
6 month visit		
N with adequate histology results	261	147
CIN 2 N (%)	25 (9.6%)	7 (4.8%)
CIN 3 N (%)	21 (8.0%)	11 (7.5%)
Cancer N (%)	0	1 (0.7%)
12 month visit		
N with adequate histology results	228	132
CIN 2 N (%)	23 (10.1%)	9 (6.8%)
CIN 3 N (%)	29 (12.7%)	9 (6.8%)
Cancer N (%)	1 (0.4%)	1 (0.8%)

^a^Number negative on the channel at follow-up divided by the number who were positive on the channel at baseline.

Three microinvasive cancers were detected among women who had undergone ablative therapy. One cancer was detected in a WHIV at the 12 month visit. The woman was persistently P3 (HPV 31, 33, 35, 53, 58) and P5 (HPV 39, 56, 66, 68) positive. A biopsy at 6 months yielded a diagnosis of cervical intraepithelial neoplasia grade 2 (CIN2) which prompted a LLETZ at 12 months, which diagnosed CIN3 and a microinvasive focus of 0.9 × 0.4 mm. The other two cancers were detected in WNoH. One woman was diagnosed on a biopsy taken at 6 months which showed a small focus of microinvasion with CIN3. She was persistently HPV 16 positive at baseline and month 6. The second WNoH had CIN2 diagnosed on a biopsy at 6 months and underwent a LLETZ at 12 months which diagnosed CIN3 with a focus of microinvasion measured at 0.2 × 0.2 mm. She was persistently P3 (HPV 31, 33, 35, 53, 58) positive.

At 6 months, 17.6% of WHIV and 12.9% of WNoH had CIN2 or worse (CIN2+) and at 12 months, 23.2% of WHIV and 14.4% of WNoH (Table 4).

#### Women who tested HPV-positive but did not meet eligibility criteria for treatment

Among 327 women who tested HPV-positive at baseline but did not meet eligibility for treatment, 278 (85.0%) were seen at 6 months and 245 (74.9%) at 12 months. At 6 months, 31.0% of WHIV had cleared all HPV and 46.7% of WNoH. At 12 months, 38.4% of WHIV and 58.6% of WNoH had cleared all HPV. The prevalence of CIN2+ at 6 months was 19.4% and 10.0% and at 12 months 19.1% and 12.9% in WHIV and WNoH, respectively (Table 5).

**Table 5:** HPV and histology results at 6 and 12 months among 327 women HPV-positive at baseline but not eligible for treatment^a^ and among random sample of 223 women HPV-negative at baseline.

	HPV-positive at baseline not treatment eligible^a^		HPV-negative at baseline	
	Women living with HIV	Women not living with HIV	Women living with HIV	Women not living with HIV
N in group at baseline	207	120	79	144
HPV results				
6 month visit				
N (% follow-up)	171 (82.6%)	107 (89.2%)	67 (84.8%)	127 (88.2%)
All 5 HPV channels				
Cleared all HPV	53 (31.0%)	50 (46.7%)	–	–
Cleared channel positive at baseline	79 (46.2%)	54 (50.5%)	–	–
New detection	26 (15.2%)	4 (3.7%)	10 (14.9%)	11 (8.7%)
Individual channel clearance				
Cleared HPV16	7/11 (63.6%)	2/2	–	–
Cleared HPV18/45	23/55 (41.8%)	14/32 (43.8%)	–	–
Cleared P3 (HPV 31,33, 35, 53, 58)	10/20 (50.0%)	15/30 (50.0%)	–	–
Cleared P4 (51,59)	13/38 (34.2%)	5/13 (38.5%)	–	–
Cleared P5 (39,56,66,68)	30/76 (39.5%)	22/37 (59.7%)	–	–
12 month visit				
N (% follow-up)	146 (70.5%)	99 (82.5%)	55 (69.6%)	109 (75.7%)
All 5 HPV channels				
Cleared all HPV	56 (38.4%)	58 (58.6%)	–	–
Cleared channel positive at baseline	69 (47.3%)	57 (57.6%)	–	–
New detection	29 (19.9%)	6 (6.1%)	3 (5.5%)	7 (6.4%)
Individual channel clearance				
Cleared HPV16	5/10 (50.0%)	1/2 (50.0%)	–	–
Cleared HPV18/45	23/51 (45.1%)	19/31 (61.3%)	–	–
Cleared P3 (HPV 31,33, 35, 53, 58)	10/17 (58.8%)	17/26 (65.4%)	–	–
Cleared P4 (51,59)	14/32 (43.8%)	4/11 (36.4%)	–	–
Cleared P5 (39,56,66,68)	40/63 (63.4%)	26/36 (74.3%)	–	–
Histology results				
6 month visit				
N with adequate histology	160	100	64	124
CIN 2 N (%)	10 (12.5%)	4 (4.0%)	0	0
CIN 3 N (%)	11 (6.9%)	6 (6.0%)	0	1 (0.8%)
Cancer N (%)	0	0	0	0
12 month visit				
N with adequate histology	141	93	53	103
CIN 2 N (%)	13 (9.2%)	6 (6.5%)	0	0
CIN 3 N (%)	14 (9.9%)	6 (6.5%)	0	0
Cancer N (%)	0	0	0	0

^a^Not treatment eligible were those with low viral loads on channels detecting HPV 16, 18, 45, 31,33, 35, 53, 58 or positive only for P4 (HPV 51, 59) or P5 (HPV 39, 56, 66, 68).

#### Women who tested HPV-negative

Among women who tested HPV-negative at baseline, a 10% random sample of 223 women were selected for follow-up and 196 (87.9%) were seen at 6 months and 165 (74.0%) at 12 months. At 6 months, 14.9% of WHIV and 8.7% of WNoH tested HPV-positive. There were no cases of CIN2 or CIN3 among WHIV and 1 case of CIN3 in WNoH. At 12 months, 5.5% of WHIV and 6.4% of WNoH tested HPV-positive. No additional CIN2+ cases were identified.

## Discussion

Operational fidelity to the HPV-SAT program was outstanding at our clinical research site. Recruitment was robust and steady. Almost all women had valid HPV test results on the first run. Almost all women stayed to receive their results. Greater than 90% of those eligible for treatment underwent thermal ablation on the same day. Task-shifting of HPV testing to a community health worker (CHW) with no prior laboratory training was successful. The program was nurse-led, with successful task-shifting of initial safety screening, sample collection, visual assessment for suitability for ablative therapy and, if suitable, performance of thermal ablation on the same day by the trained study nurse. Thermal ablation resulted in only minor complications in a small proportion of those treated. In sum, HPV-SAT successfully managed a round of screening at a single-visit, dramatically reducing the numbers of women who needed to be up-referred to specialist colposcopy services which are over-burdened in this setting.

South Africa has the highest rate of people living with HIV globally, with approximately 8.2 million in 2021, and cervical cancer screening services need to take into account the special needs of this group. The geography and variation in HIV prevalence influences screening outcomes, due to the different natural history of HPV infection in people living with HIV^9,10^. Failure of either ablative or excisional treatment of cervical cancer precursors has been well-documented in WHIV, due to decreased clearance of HPV infection, increased progressive disease and decreased regression in WHIV^11^. The proportion of WHIV in our study (44%) nearly doubles the 21% expected HIV prevalence in adults living in Khayelitsha^12^. This overrepresentation of WHIV in our study may reflect that people living with HIV access health services more often than women who are uninfected. Paradoxically, women without HIV infection are an under-screened population in our setting and special efforts must be made to ensure that they are adequately brought into services.

The potential of POC testing to improve health care in LMICs has been widely discussed^13^. In our study, using a CHW to perform Xpert HPV testing on-site avoided logistical challenges with transporting samples to a central laboratory, loss of time, and the costs of transportation and laboratory-trained technicians. Most importantly, however, on-site testing enabled women to receive their results at the same visit, avoiding inevitable delays and attrition with results given at a subsequent visit. POC testing enabled the research clinical team to provide post-test counselling to HPV-positive women, an important intervention for comprehensive screening and treatment. A recent study from Peru reported in-depth interviews with women with a positive HPV test. Most women reported experiencing fear after receipt of a positive HPV test, particularly regarding how they became infected and by associating HPV infection with cancer, and death. This was followed by relief knowing that simple and fast treatments could be performed as an outpatient at primary care level. Counselling about the virus and expectations of treatment significantly reduced anxiety and fear among the participants^14^.

POC testing will, however, require significant investment including, capital outlay for equipment as well as establishing reliable supply-chain management systems, commodities security and data management systems that may be less robust at the outset than utilizing established centralized laboratory systems. Reliable electricity supply is also needed which is a challenge in many LMICs, especially in South Africa. Our study was based at a clinical research site with research staff dedicated to screening, a GeneXpert instrument dedicated to HPV testing that did not need to be shared with other services, and adequate infrastructure and facilities. These aspects of HPV-SAT will require careful planning for sustainable integration within primary care facilities where patients with a range of health conditions are seen.

Most women were informed of their test result within one and a half hours from sample collection. For most patients, the waiting time cannot be shortened further—Xpert HPV requires 60 min run time in the machine and the additional 30 min accounted for moving the samples from the exam room to the onsite laboratory, sample processing and loading, and results tabulation before returning results to the patient. Given available staff on site, one four module GeneXpert instrument was adequate for the number of women who could be seen daily. Instruments with larger capacities would allow for a higher testing volume with increased clinical staff capacity. The observation that most patients waited for return of their results and treatment, if needed, can be interpreted that the same-day, HPV-SAT is acceptable to patients. However, we did not directly inquire if the waiting time was acceptable. In addition, this wait time does not include time spent prior to sample collection or the time required for counselling and treatment, if necessary, after the HPV result was conveyed. We made provision for a waiting area with refreshments and made sure, through counselling and education, that women understood what was involved in a round of screening. In routine services it may be difficult to accommodate such long visit durations and to provide waiting area space. A possible solution could be to conduct informed consent, counselling and education at one visit, and then have a second visit for HPV testing and treatment. However, such an approach would add another layer of complexity and increase the likelihood of attrition. Adequate linkage to care is crucial when testing and treatment occur in different locations^15^.

An important outcome of using our modified definition of a positive HPV test (high viral load on the three channels that detect types 16, 18, 45, 31, 33, 35, 52, 58 or multiple types) to define eligibility for treatment was that it reduced by almost 40% the numbers of women who needed treatment. This benefit, however, came at the cost of relatively high rates of HPV persistence and pre-cancerous disease in those who were HPV-positive without meeting our modified criteria (almost 20% of WHIV in this category had histologically confirmed CIN2+ 12 months after initial screening). While our approach reduces overtreatment, in settings with such high rates of HIV-positivity, HPV prevalence and cervical disease, restricting treatment to only those with a high viral load, results in women at lower but still high risk of disease being left untreated. In a situation where treatment resources are scarce, our approach is justified as it targets scarce treatment resources to the highest risk women. However, where treatment resources permit, widening the net may be more justified. Rationing treatment should be driven by resource availability and the ability to provide high levels of care to screened women. The specific cut-offs selected for programs should be based on a pragmatic approach which takes the context in which the program is offered and the resources available into account.

Our data support the very high negative predictive value of a negative HPV test, in women both with and without HIV infection and support the notion of a wider interval for rescreening of at least 5 to 10 years, which in itself will result in reduced burden on screening resources.

The initial nurse pre-screen examination detected four cancers. One of these four was HPV-negative and would have been missed by an HPV screening program that did not include this safety net. Although the purpose of screening is to detect pre-cancerous disease, in under-screened populations like the one studied here, programs need to be cognizant of the importance of detecting existing cancers and facilitating pathways into care. We recommend that screen-and-treat programs retain and use the clinical skills of the health care providers including visual inspection to detect clinically visible cancers and appropriate history taking to elicit symptoms related to genital tract disease.

Consistent with prior studies^16–18^, no serious adverse events or hospitalisations were recorded following thermal ablation supporting the safety of this procedure by a trained nurse.

Rates of histologically confirmed disease post-treatment were relatively high (23% CIN2+ in WHIV and 14% in WNoH) indicating that thermal ablation was only partially successfully in treating precursor lesions. As we did not perform histological assessment of women at baseline, it is not possible to calculate the cure rate. Nevertheless the rate of 86% of women without HIV being free of histologically confirmed disease is identical to the cure rate reported in a meta-analysis for studies conducted in low and middle income studies although lower than the 94% cure rate they report for studies from higher income countries^19^. A study performed in Honduras, not included in the meta-analysis, reported a higher cure rate (94%) based on being free of CIN2+ on histology^20^. Cure rates for women living with HIV were not reported out separately in the meta-analysis^19^.

Our protocol defined clearance of HPV as the primary marker of treatment effectiveness and at the 12 month visit, only 39% of WHIV and 65% of WNoH were clear of all HPV. Even among women without HIV, these clearance rates are lower than have been reported from elsewhere. In a study from Cameroon, 84% of women treated with thermal ablation had cleared their HPV infection by 12 months^21^. In a multisite study in rural China, 86% of treated women had cleared all high risk HPV types after thermal ablation, although rates were lower for clearance of HPV16/18 (68%)^22^. A randomized trial of thermal ablation vs. cryotherapy also conducted in China reported rates of HPV clearance exceeding 80% in both groups^23^.

Reasons for the low clearance rates in our study are unknown but it is interesting that if clearance is defined strictly as clearance of a channel present at baseline, clearance rates are higher. At 12 months, 59% of WHIV and 69% of WNoH were clear of the infection they had at baseline compared to 39% of WHIV and 65% of WNoH defining clearance as being free of all high-risk HPV. The low rates of HPV clearance in our study, particularly in WHIV, may in part be explained by newly acquired infections or activation of latent infections. Our study recruited a largely unscreened or under-screened population and the prevalence of HPV, even among women without HIV, was high. We further restricted ablative therapy to those with higher viral load on the most oncogenic HPV genotypes mostly likely selecting those most challenging to treat. Larger lesions, poorer immunity due to co-morbid infections such as tuberculosis, HIV, high levels of stress and poor-quality nutrition related to poverty and low socio-economic status are associated with increased risk of pre-cancerous lesions^24^. All the women recruited to our study were South African women living in the peri-urban settlement of Khayelitsha where women have a high rate of exposure to these modifiable risk factors. Whether exposure to these risk factors explains the high rates of persistence of infection and disease post thermal ablation in this study will require further research.

Our study classified more than 90% of those meeting HPV eligibility criteria for treatment as being suitable for ablative therapy. In a study of 4285 women in Papua New Guinea (PNG)^25^, 647 women tested positive for HPV using Xpert HPV, of whom 603 were considered eligible for same day ablation (94%) and 602 underwent treatment, a similar proportion as in our study. It may be that over 90% suitability for ablative treatment in both our and the PNG study is too high, particularly among unscreened populations of women, with high levels of preinvasive and invasive cervical disease. However, similar proportions of screened women were suitable for same day thermal ablation in studies performed in Malawi, 93.3%^26^ and Cameroon, 90.9%^27^, both low resource settings where the majority of women are unscreened.

The Scale-Up Project conducted in Guatemala, Nicaragua and Honduras used careHPV testing with triage either by Pap or VIA, and, if triage-positive, women were referred for ablative therapy^28^. This study recruited 231,741 women in three countries with an overall HPV-positivity rate of 13.6% and the majority tested using self-sampling. There was a delay of one month in receiving careHPV results which contributed to loss to follow up. However, 84.7% of women in Guatemala, 67.1% in Nicaragua and 58.8% in Honduras were suitable for and underwent ablative therapy, initially using cryotherapy and later using thermal ablation^28^.

## Strengths and limitations

This study has several strengths: Our clinical research site, co-located in a real-world primary health care setting, serves a largely unscreened population of women in the targeted age group of 30–65 years. All women were recruited from Khayelitsha, an area typical of low resource settings with high levels of co-morbid conditions and inadequate health care services. Study procedures were nurse-driven and performed on-site but with back-up of a study medical officer when required. HPV testing was performed by a CHW using a validated POC, HPV DNA PCR assay. The CHW was quickly up-skilled and proved capable of performing the test and undertaking the interpretation for eligibility for treatment. The ability to perform on-site HPV testing enabled over 90% of women eligible and suitable for ablation to receive treatment at the same visit. Linking screening to treatment is a major advance in cervical cancer prevention and will lead to greater coverage, efficiency and effectiveness of screening programs.

Limitations of the study include the lack of histological sampling at baseline to determine prevalence of CIN2+ and hence our inability to estimate cure rates. We did not perform an in-depth acceptability evaluation, other than documentation of adherence to protocol. Future studies of screen-and- treat warrant a deeper assessment of women’s experiences and whether these pose a barrier to establishing services.

## Conclusions

Our study provides strong evidence that screening with a point-of-care HPV test and treating women using thermal ablation at the same visit has potential to be safe and feasible in a low resource primary care setting but further implementation studies are required.

We have identified limitations to the approach of restricting treatment to only those with high viral load on certain HPV genotypes. While this approach has the advantage of reducing the number of women requiring treatment as well as the rate of overtreatment, rates of CIN2+ in women with HPV at lower viral loads was still quite high and these women are at risk of developing cervical cancer. The decision to use such modified cut-offs, and thereby restrict treatment, should be based on available resources and the context in which screening is implemented, balancing the trade-offs.

The relatively high persistence of HPV infection and CIN2+ at 6 and 12 months post treatment in women in our study highlights the need for more research to improve treatment options. Currently available options (i.e. thermal ablation, cryotherapy or excisional procedures) have major limitations and constraints. Other treatment options and/or adjunctive therapies urgently need to be investigated, including therapeutic vaccination.

This demonstration study supports the feasibility and safety of HPV-SAT for cervical cancer prevention. HPV-SAT will add value to other efforts to eliminate cervical cancer as a public health problem. Implementing HPV-SAT should be incremental, with careful attention to the infrastructure and ecosystem required for successful and efficient outcomes and full integration into routine primary health care services.

## Methods

The study was implemented at a clinical research site co-located with a primary health care facility in Khayelitsha, outside Cape Town, South Africa. All clinical staff were employed by the research study. A single-visit, HPV-based screen-and-treat program was implemented which recruited women of known HIV status aged 30–65 years from the surrounding community. Point-of-care Xpert HPV was the primary screening test, with a positive result defined according to a modification based on HPV genotype and viral load restriction, previously validated^7,8^. A positive HPV screen using the modified definition, was followed by a trained nurse visually assessing the woman for suitability for ablative therapy and, if suitable, thermal ablation was performed on the same day. The study was approved by the Human Research Ethics Committee of the University of Cape Town and the Institutional Review Board of Columbia University. All research was performed in accordance with relevant guidelines and regulations of these committees and the Declaration of Helsinki. All participants provided written informed consent.

### Description of the screen-and-treat approach

Figure 1 shows the steps in the screen-and-treat cascade. As part of obtaining written informed consent, we conducted pre-screening education and counselling that included the rationale for cervical cancer screening, the procedures involved, and the expectations for follow-up and treatment, if needed. Eligible participants were women aged 30–65 years, with no history of hysterectomy, no severe gynecologic symptoms suggestive of cervical cancer, not pregnant, documented HIV status, and willing and able to provide written informed consent. Women of unknown HIV status were rapid-HIV-tested according to guidelines. All pre-menopausal women not on reliable contraception were tested for pregnancy.

A trained study nurse performed a gynecologic examination, visually inspecting the lower anogenital tract and cervix without acetic acid for clinically detectable cancers and other markers suspicious for cancer. This examination was designed as a safety net for possible HPV-negative cancers. All women flagged as suspicious for cancer or with other concerning conditions, such as polyps, infection, atrophy or poor visibility of the cervix, were reviewed by an onsite study doctor regardless of HPV results. Next, the nurse collected a sample from the cervix using a plastic spatula and a cervibrush into 20mls of Preservcyt^®^ThinPrep^®^PapTest solution (Hologic, Danbury, CT) for HPV testing.

The nurse-collected specimen was HPV tested using Xpert HPV (Cepheid, Sunnyvale, CA) on-site. Xpert HPV is a cartridge-based real-time PCR test run on the GeneXpert platform with results obtained in 1 h. A 4-module GeneXpert instrument was dedicated to the study. The system requires consistent electricity supply and linkage to a laptop computer, and printer. An inverter was installed to maintain power to the instrument given frequent interruptions to the electricity supply. Xpert HPV detects 14 high-risk HPV types with results reported in 5 separate channels: HPV16 (P1); HPV18,45 (P2); HPV31,33,35,52,58 (P3); HPV51,59 (P4); and HPV39,56,66,68 (P5). Xpert HPV is CE- marked since 2014 and received WHO prequalification in 2017. The test was validated by the National Health Laboratory Service (NHLS) of South Africa in 2016. The tests were run by a trained Community Health Worker (CHW) with no previous laboratory training and women were asked to wait for their results. The CHW received 3 days of training in running the assay, maintaining quality control and classifying the women into eligible or not eligible for treatment. Other members of the study team were also trained to perform these steps in circumstances when the designated CHW was absent. The time when the sample was collected was recorded on the participants’ record as well as the time when the result was returned to them to calculate the amount of time that was required to wait for the HPV result.

Using standard output from Xpert HPV, we modified the definition of a positive result to identify women needing treatment as validated in our previous study^8^. Previously, we found that sensitivity of HPV testing to detect precursor lesions was minimally affected and specificity substantially improved if the definition of a positive result was restricted only to channels P1, P2 and P3 on Xpert HPV detecting HPV genotypes 16, 18, 45, 31, 33, 35, 52, 58^7,8^. We demonstrated further that with minor trade-offs in sensitivity, specificity could be further improved by restricting to lower cycle threshold (CT) values (i.e. higher viral load) on these three channels, with slightly different CT cut-offs identified for WHIV and for women not living with HIV (WNOH)^8^. For this demonstration study, we developed a manual look-up table for the CHW running the HPV test to classify women into one of three categories: (1) HPV-positive according to our modified interpretation and therefore in need of treatment, (2) HPV-positive but not meeting our modified criteria and (3) HPV-negative. For women who were positive on only one of the three channels of interest, we used the CT cut-off identified in our validation study that yielded a sensitivity of ~ 84% and a specificity for WHIV of 78% and WNOH of 92%. These were for WHIV 34.5, 29.2 and 35.1, and for WNOH 39.2, 28.1 and 34.9 for P1 (HPV16), P2 (HPV18,45) and P3 (HPV 31, 33, 35, 52, 58) respectively. In addition, we included all women positive on more than one of the three channels of interest regardless of CT value (Appendix A).

Women who met the modified criteria for a positive result underwent a second gynecologic examination by the study nurse with the application of 3–5% acetic acid. The cervix was assessed for suitability for ablative therapy. Criteria for suitability for ablative treatment in this group were that (1) the cervix was accessible (2) there was no evidence or suspicion of invasive cancer (3) the squamocolumnar junction was visible (4) there was no marked inflammation, infection or severe atrophy and (5) any visible aceto-white lesion covered less than 75% of the cervix and did not extend into the endocervical canal. Nurses could refer women to the on-site doctor if they were uncertain about their interpretation of the findings or if they considered the woman needing a higher level of care. The on-site doctor had the option of referring women back to the nurse if women were deemed suitable for same-day ablative therapy. Women were offered same-day ablative treatment unless a scheduled appointment was required.

Study nurses used thermal ablation to perform ablative therapy. Thermal ablation uses a heated probe to destroy cells and tissues on the surface of the cervix, at a temperature of 100–120 °C and applied for 20 s, over the acetowhite lesion. Repeated applications of the probe are permitted. No local anaesthetic is required. The device is battery-driven [Cure Medical Global, formerly Liger Medical LLC, Lehi, UT]. Any immediate complications were noted, and women were advised to return to the site or to their nearest primary care clinic if they developed any untoward symptoms such as pain, bleeding or an offensive discharge. While women were encouraged to return to the site for any complications, they were also given a letter that included relevant details about the screening and treatment procedure to present to other facilities. Women were encouraged to report to the nearest health facility emergency service in case of complications, especially bleeding, occurring afterhours, nights, weekends or public holidays. All women were also counselled to abstain from sexual intercourse or to use a condom if abstaining was not possible for 4 weeks after the procedure.

Women who met the modified criteria for a positive HPV screen, but were not suitable for ablative therapy, were referred to the on-site doctor for assessment. If Large Loop Excision of the Transformation Zone (LLETZ) was indicated, it was usually performed by an on-site study doctor. If the on-site doctor determined that further investigations or other interventions were needed, the woman was referred off-site to secondary or tertiary-level services.

### Evaluation

The primary outcome measured operational fidelity to the screening algorithm and quantification of the proportions of women proceeding through the steps in the screening cascade (Fig. 1).

Figure 2 shows the evaluation design schema. All women who tested HPV positive, whether or not they met the modified treatment criteria and a 10% random sample of women who tested HPV-negative based on standard cut-offs were asked to return at 6 and 12 months to ascertain HPV clearance. The remainder of the women who were HPV-negative were discharged and counselled to return in 10 years for repeat screening as per South African National Screening Policy. At the follow-up visits, a clinician-taken sample was tested on-site with Xpert HPV. All women underwent a colposcopic assessment and histological sampling. Aceto-white lesions were biopsied and if no lesion visible, an endocervical curettage was performed. Histological specimens were reviewed in a private pathology laboratory in Cape Town. All slides of women classified as cervical intraepithelial neoplasia grade 2 or worse (CIN2+) on only one of the two visits were re-reviewed and the second diagnosis utilized. In addition, cases of CIN2+ had to have positive HPV test results at the time the sample was taken to be classified as a true case.

Women who had undergone ablative therapy were asked at the 6- and 12-month visits about any complications they had experienced following treatment. The protocol defined the primary marker of ablative treatment effectiveness as the repeat HPV testing with Xpert HPV at 6 and 12 months post-treatment. Histological sampling was not performed before treatment precluding evaluation of the effectiveness of treatment based on this marker. Confirmed CIN2+ at 6 and 12 months were used as secondary markers of treatment failure.

In women who tested HPV-positive on conventional cut-offs but did not meet modified criteria and in the random sample of women who tested HPV-negative, confirmed CIN2+ was used a marker of the magnitude of disease missed by the screening algorithm.

### Statistical considerations

We aimed to recruit 3000 women over 18 months, 1000 of whom were estimated to be WHIV. We estimated that ~ 480 women would meet the treatment HPV criteria, ~ 300 women would test HPV-positive but would not meet our criteria and that ~ 250 HPV-negative women would be randomly-selected for follow-up, leading to 1030 women scheduled for follow-up visits. The sample size was determined based on logistical, human and resource constraints with the number of women we could enrol, screen and follow-up.

Descriptive statistics for categorical (i.e., frequencies and proportions) and continuous variables were calculated as appropriate (i.e., means and standard deviations, or medians and interquartile ranges [IQR]). Statistical analysis was done in SAS 9.4 (Cary, NC).

### Supplementary Information

Supplementary Information.

## Data Availability

Deidentified data are available from the corresponding author to researchers with an approved protocol who complete a data use agreement.
