# Supplementary material for: Point-of-care testing with Xpert HPV for single-visit, screen-and-treat for cervical cancer prevention: a demonstration study in South Africa
Source: Sci Rep. 2023 Sep 27;13:16182. doi: 10.1038/s41598-023-43467-2 (PMC10533854; doi:10.1038/s41598-023-43467-2)

**Appendix 1**

Page 1 shows an example print-out from Xpert HPV indicating the Analyte Name (channel of interest), the cycle threshold (Ct) value for each channel and the summary result for each channel.

Page 2 shows the steps to be followed in interpreting the Xpert HPV result to classify a women with HIV has being eligible for treatment based on the HPV algorithm.

Page 3 shows the steps to be followed in interpreting the Xpert HPV result to classify a women without HIV has being eligible for treatment based on the HPV algorithm.

Page 4 shows a look up table of cycle threshold values for the three channels of interest in defining women with and without HIV as meeting eligibility criteria for treatment.

# Test Report

Sample ID: G1027
Test Type: Specimen
Sample Type: [redacted]

## Assay Information

| Assay | Assay Version | Assay Type |
|---|---|---|
| HPV HR AND GENOTYPE RUO ASSAY | 2 | Research Use Only |

Test Result: HPV 16 NEG;
HPV 18_45 POS;
OTHER HR HPV POS

Ct value

## Test and Analyte Result

| Analyte Name | Ct | EndPt | Analyte Result | Probe Check Result |
|---|---|---|---|---|
| SAC | 36.8 | 45.0 | NA | PASS |
| HPV 16 | 0.0 | 0.0 | NEG | PASS |
| HPV 18_45 | 30.9 | 322.0 | POS | PASS |
| P3 | 26.6 | 366.0 | POS | PASS |
| P4 | 0.0 | 7.0 | NEG | PASS |
| P5 | 0.0 | -3.0 | NEG | PASS |

[illegible]

Analyte name

Analyte result

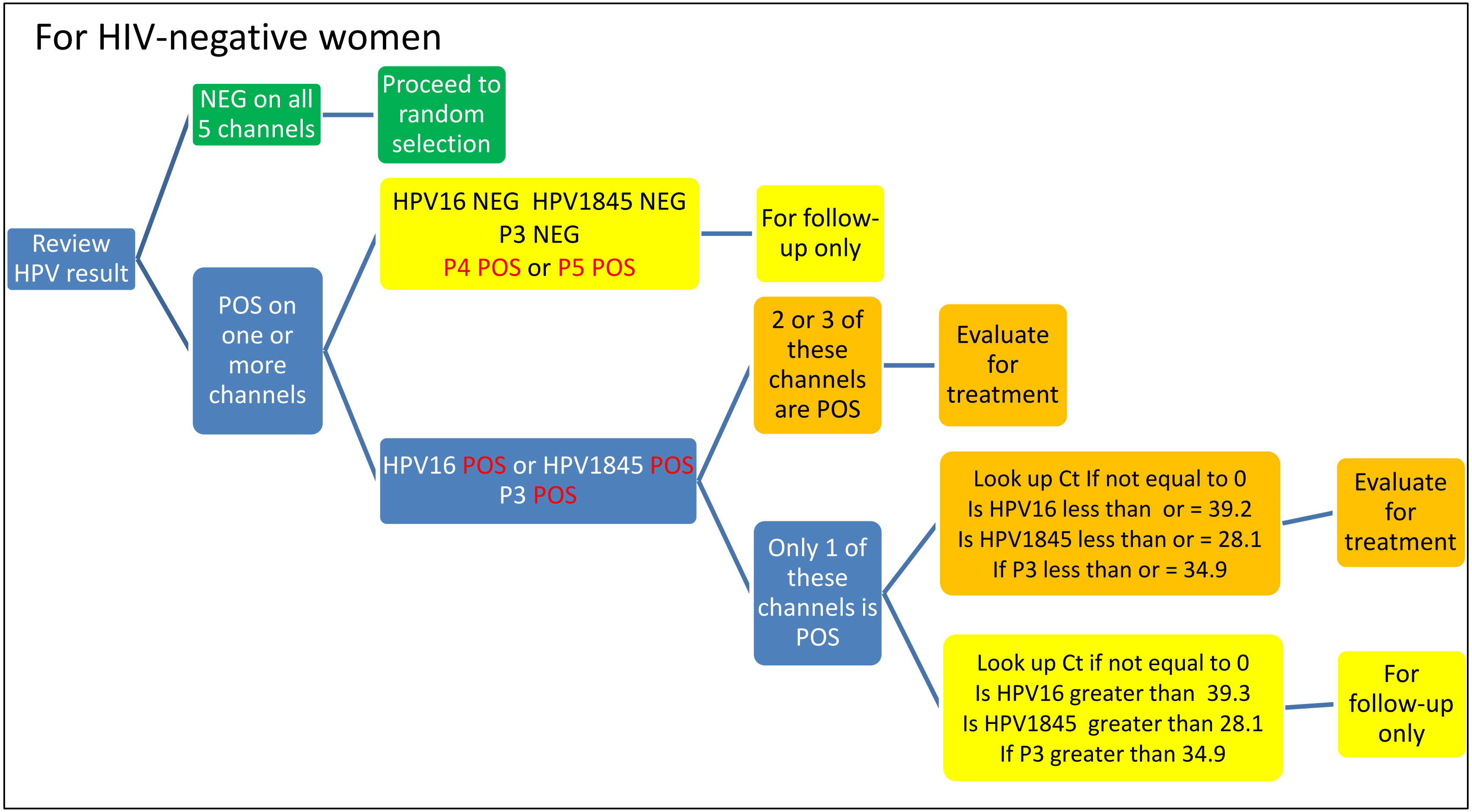

For HIV-negative women
Review HPV result
NEG on all 5 channels
Proceed to random selection
POS on one or more channels
HPV16 NEG HPV1845 NEG P3 NEG P4 POS or P5 POS
For follow-up only
HPV16 POS or HPV1845 POS P3 POS
2 or 3 of these channels are POS
Evaluate for treatment
Only 1 of these channels is POS
Look up Ct If not equal to 0 Is HPV16 less than or = 39.2 Is HPV1845 less than or = 28.1 If P3 less than or = 34.9
Evaluate for treatment
Look up Ct if not equal to 0 Is HPV16 greater than 39.3 Is HPV1845 greater than 28.1 If P3 greater than 34.9
For follow-up only

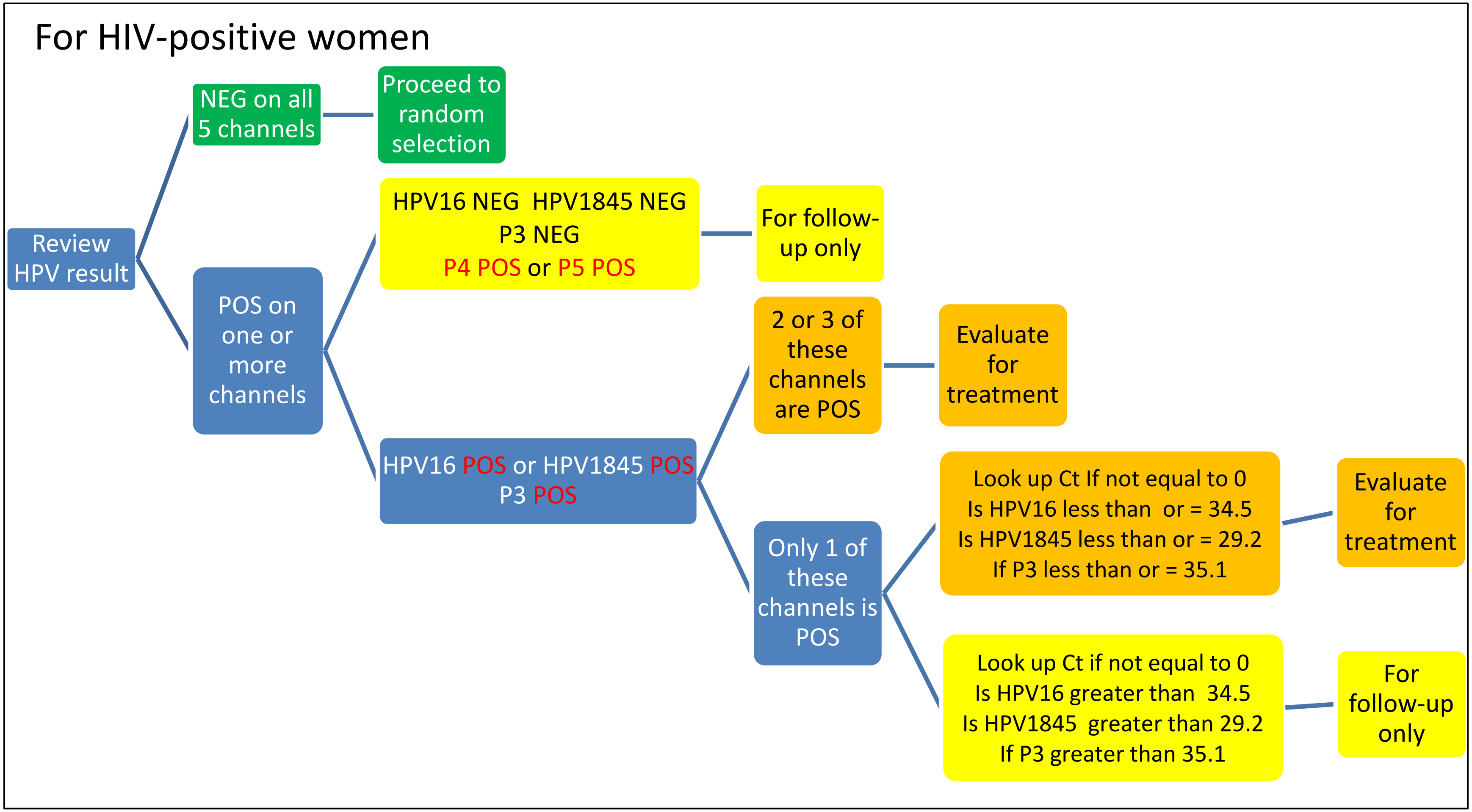
For HIV-positive women
Review HPV result
NEG on all 5 channels
Proceed to random selection
POS on one or more channels
HPV16 NEG HPV1845 NEG P3 NEG P4 POS or P5 POS
For follow-up only
HPV16 POS or HPV1845 POS P3 POS
2 or 3 of these channels are POS
Evaluate for treatment
Only 1 of these channels is POS
Look up Ct If not equal to 0
Is HPV16 less than or = 34.5
Is HPV1845 less than or = 29.2
If P3 less than or = 35.1
Evaluate for treatment
Look up Ct if not equal to 0
Is HPV16 greater than 34.5
Is HPV1845 greater than 29.2
If P3 greater than 35.1
For follow-up only

If 2 or 3 of HPV16, HPV1845 or P3 are positive = For treatment
If 1 of HPV16, HPV1845 or P3 is positive, look at the Ct value
If smaller than or equal to the cut-off shown → For treatment
If bigger than the cut-off shown → For follow-up

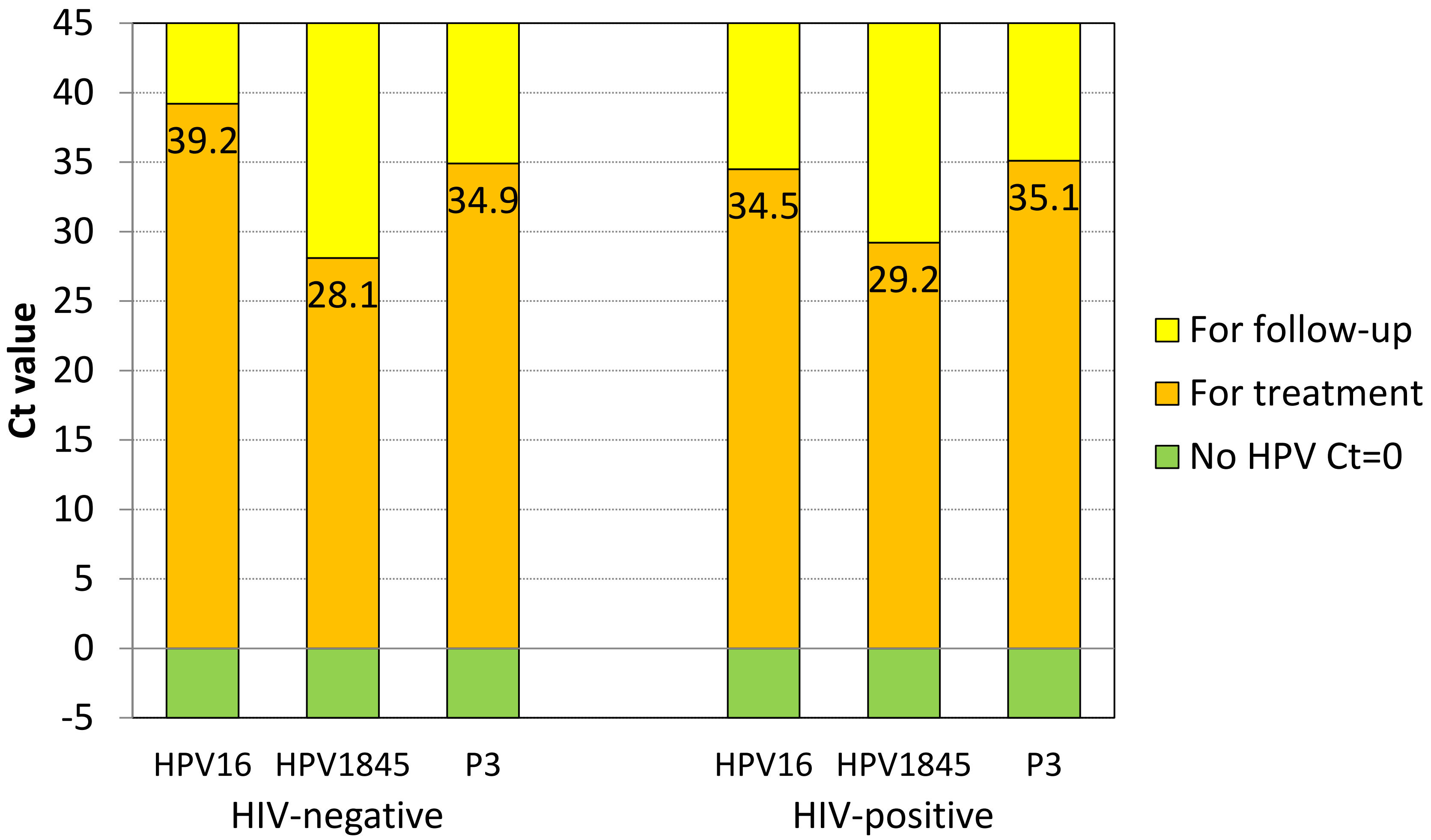

Supplement: Supplementary file 1 — Supplementary Information. [file 41598_2023_43467_MOESM1_ESM.pdf]
